# A High-Fat Diet Induces Oxidative Stress in OPA1^+/−^ Mouse Cortices: A Critical Double Challenge

**DOI:** 10.3390/antiox14070876

**Published:** 2025-07-17

**Authors:** Camille Champigny, Marlène Botella, Djamaa Atamena, Sébastien Bullich, Corentin Coustham, Bruno Guiard, Pascale Belenguer, Noélie Davezac

**Affiliations:** 1 Got-It Team, RESTORE—University of Toulouse, CNRS ERL5311, EFS, INP-ENVT, Inserm U1031, Bâtiment INCERE, 4bis avenue Hubert Curien, 31100 Toulouse, France; camille.champigny@inserm.fr (C.C.); corentin.coustham@inserm.fr (C.C.); 2 Minding Team, Research Center on Animal Cognition (CRCA), Center of Integrative Biology (CBI)—University of Toulouse, CNRS, 31067 Toulouse, France; marlene.botella@univ-tlse3.fr (M.B.); djamaa.atamena@univ-tlse3.fr (D.A.); pascale.belenguer@univ-tlse3.fr (P.B.); 3 Remember Team, Research Center on Animal Cognition (CRCA), Center of Integrative Biology (CBI)—University of Toulouse, CNRS, 31067 Toulouse, France; sebastien.bullich@mpfi.org (S.B.); bruno.guiard@univ-tlse3.fr (B.G.)

**Keywords:** cortices, antioxidant defenses, mitochondria, OPA1, neurodegenerative disease

## Abstract

A high-fat diet (HFD) has significant effects on health, leading to cardiovascular, metabolic, neurodegenerative, and psychiatric conditions and contributing to obesity and type 2 diabetes. Mitochondria, essential for energy production and oxidative metabolism, are adversely affected by a HFD, causing oxidative stress and impaired cellular function. Mutations in the *OPA1* (OPtic Atrophy 1) gene, crucial for mitochondrial dynamics and functions, are responsible for dominant optic atrophy (DOA), a mitochondrial neurodegenerative disease associated with increased reactive oxygen species (ROS). The expressivity of DOA is highly variable, even within the same family. This suggests that both modifying genetics and environmental factors could influence the penetrance of the disease. We previously demonstrated that genetic background modulates DOA expressivity and now ask if this is also the case for external cues. We thus explore how OPA1 deficiency interacts with HFD-induced metabolic disturbances, hypothesizing that long-term HFD consumption impairs brain mitochondrial function and disrupts oxidative metabolism. OPA1^+/−^ mice were thus subjected to a HFD for a period of 12 weeks, and ROS levels and the expression of antioxidant genes were evaluated by Western blot and spectrophotometry. Cortices from high-fat diet-fed OPA1^+/−^ mice showed lower aconitase activity than those of their wild-type (WT) litter mates, indicative of an unbalanced increase in mitochondrial ROS. Accordingly, OPA1^+/−^ mice present lower levels of the antioxidant enzyme superoxide dismutase 2 compared to WT mice. Therefore, this study (i) reveals the onset of oxidative stress in brain cortices from OPA1^+/−^ mice challenged with a HFD, (ii) shows that diet is a modifying factor for DOA, and (iii) suggests that food control could be used to moderate the severity of the disease.

## 1. Introduction

Lifestyle, and especially diet, has a significant impact on overall health. A high-fat diet (HFD), characterized by excessive lipid consumption, has deleterious consequences associated with the development of pathologies such as cardiovascular, metabolic, neurodegenerative, and psychiatric diseases [1,2,3]. A HFD, and particularly the D12451 formulation, is commonly used to induce obesity and metabolic syndrome in mice. Quantitatively, the D12451 diet provides about 4.73 kcal/g, with 24% of its energy from protein, 41% from carbohydrates, and 45% from fat. This composition contrasts sharply with standard rodent chow, which generally contains 10–15% fat by energy. The primary fat source in the D12451 diet is usually lard, which provides a high level of saturated fats. The high-fat content in the D12451 diet leads to significant weight gain, increased adipose tissue mass, and alterations in glucose and lipid metabolism in mice. This dietary manipulation is crucial for studying the pathophysiological mechanisms underlying obesity, insulin resistance, neuroinflammation, and other metabolic disorders [4,5,6,7]. In animal and in vitro models, a lot of saturated fatty acids are associated with neuroinflammation, neuronal loss, and mitochondrial dysfunctions [8,9].

Mitochondria, crucial organelles within cells, play a central role in oxidative metabolism and energy production. Their dysfunctions are involved in different pathologies, are there are no direct therapies to date [10,11]. Moreover, their plasticity is directly linked to lifestyle factors, including nutrition and exercise [12]. Thus, understanding the mechanisms underlying mitochondrial functions is crucial in biomedical research, notably to identify new therapeutic interventions for lifestyle-related metabolic disorders [13,14].

In recent years, the OPA1 (OPtic Atrophy 1) gene has garnered considerable attention due to its impact on mitochondrial functions, as well as its connections with oxidative metabolism dysfunction. The OPA1 gene encodes the OPA1 mitochondrial protein, which is found in the mitochondrial intermembrane space associated with the inner mitochondrial membrane. Research using common genetically modified cell lines, such as HeLa cells, has revealed that OPA1 plays multiple roles, including inner membrane fusion, cristae organization, mitochondrial DNA maintenance, mitochondrial energetics regulation, and anti-apoptotic effects [15,16,17]. OPA1 mutations in humans can lead to neurodegenerative disease, specifically dominant optic atrophy (DOA) [18]. DOA is characterized by a moderate to severe loss of visual acuity, with insidious onset in early childhood. Moreover, severe multisystemic disorder, associated with specific OPA1 mutations, is observed in 30% of patients (DOA+ syndrome). Haploinsufficiency is the main pathological mechanism of isolated DOA, while DOA+ could be the consequence of negative dominance. The incomplete penetrance and great variability of DOA expressivity within and between families suggest that genetic and/or environmental factors could influence the severity and progression of the disease. Interestingly, we recently demonstrated that genetic modifying factors modulate the severity of the disease in a DOA mouse model [19]. However, so far, the contribution of environmental factors to DOA expressivity has not been studied. Several susceptibility factors have been identified for another optic neuropathy, Leber Hereditary Optic Neuropathy (LHON), caused by mutations in the mitochondrial respiratory complex 1 (MRC1). These include excessive tobacco and alcohol consumption and exposure to specific toxins and drugs affecting mitochondria [20]. This suggests that the effects of primary mutations on gene coding mitochondrial protein, such as OPA1 or subunits of MRC1, can be worsened by external cues weakening mitochondria.

Several models of DOA have underlined the role of oxidative stress in the etiology of the disease, similar to what has been proposed for many neurodegenerative diseases, such as Alzheimer’s disease, Parkinson’s disease, and LHON [21,22,23,24]. Two invertebrate models of DOA have highlighted the significant generation of reactive oxygen species (ROS) associated with OPA1 dysfunction [18]. Our pioneering work showed that this is also the case in mammals. We indeed showed in vitro in Hela cells and in cortical neurons in primary culture, as well as in mouse cortices, that the inactivation of OPA1 induces an oxidative imbalance by increasing ROS levels [15,25,26]. Accordingly, OPA1 knockdown in siRNA-transfected HEK-293 cells was shown to increase ROS generation in mitochondria [27], and hypertensive transgenic OPA1^+/−^ mice displayed an increase in ROS compared to hypertensive wild-type (WT) mice in vascular smooth muscle cells [28].

Altogether, these data prompt us to examine whether challenging mitochondrial metabolic disturbances induced by an unbalanced diet may interact with OPA1 inactivation. It is indeed well known that a HFD disrupts the mitochondrial oxidative metabolism in several tissues, including the brain, leading to oxidative stress and alterations in cellular energy production [29,30]. Our working hypothesis was that the prolonged consumption of a HFD has a detrimental effect on mitochondrial function in the brain, which in turn promotes alterations in oxidative metabolism, with an impact on phenotypes associated with OPA1 dysfunction.

## 2. Materials and Methods

### 2.1. Animals and Diet Composition

Mice were bred and maintained at the Center of Integrative Biology animal facility (authorization number 31-555-011) under standard housing conditions, including a 12 h light/dark cycle, with food and water provided ad libitum. All procedures complied with European Union guidelines (2010/63/EU). According to these regulations, ethical approval is only required for experimental procedures involving animals. In accordance with Article R214-89 of the French Rural Code, euthanizing animals solely for the purpose of organ or tissue collection does not constitute an experimental procedure. The mice used in this study (OPA1^329–355del^ C57BL/6 J abbreviated OPA1^+/−^), derived from the B6; C3-Opa1^329–355del^ strain, were housed in groups of five in specific pathogen-free, temperature-controlled barrier facilities [19]. Wild-type littermates served as controls and were consistently included in experiments. Genotyping was systematically performed to confirm group assignments. At 10 months of age, both wild-type and mutant mice were administered a high-fat diet (D12451, 40% animal fat; Research Diets Inc., New Brunswick, NJ, USA) for a duration of 12 weeks. Upon sacrifice, after these 12 weeks, the cortical regions of adult brains were isolated and separated from the hippocampus and cerebellum. Each of these regions was snap-frozen in liquid nitrogen and stored at −80 °C. Cortices were then thawed on ice, and 20 mg of tissue was collected and processed for immunoblotting experiments.

### 2.2. Metabolic Parameters: Intraperitoneal Glucose Tolerance Test (ipGTT)

On the day of the experiment, animals were individually housed, weighed, and subjected to a 5 h fasting period (from 9 a.m. to 2 p.m.) with free access to water. This fasting protocol was chosen based on prior evidence indicating that short-term fasting is optimal for evaluating insulin sensitivity while minimizing disruption to overall metabolism [31]. Blood glucose levels were measured from tail-tip blood samples using an Accu-Chek Performa glucometer (Roche, Boulogne-Billancourt, France) at baseline (0 min) and at 15, 30, 45, 60, 90, and 120 min following intraperitoneal glucose administration (2 g/kg at weeks 0 and 6, or 1 g/kg at week 11). The area under the curve (AUC) was calculated using the coordinate axis baseline set at 100, serving as an index of glucose tolerance.

### 2.3. Metabolic Parameters: Plasma Insulin Levels

To assess plasma insulin levels, blood samples were collected from the tail-tip immediately prior to the ipGTT using heparinized capillary tubes (Microvette CB 300 K2E, Sarstedt, Marnay, France). Samples were centrifuged at 10,000 rpm for 10 min, and the resulting plasma was harvested and stored at −80 °C until further analysis. Insulin concentrations were determined using the AlphaLISA assay, in accordance with the manufacturer’s instructions (Human Insulin Kit, catalog #AL204C; PerkinElmer, Villepinte, France).

### 2.4. Metabolic Parameters: HOMA-IR

Homeostasis model assessment of insulin resistance (HOMA-IR) scores were calculated using fasting glucose and insulin concentrations obtained after a 5 h fast. The formula applied was

$$HOMA-IR=\frac{fasting\;blood\;glucose\;(mg/dL)\times fasting\;insulin\;(ng/mL)}{405}$$

An HOMA-IR value equal to or greater than 2.8 was considered indicative of insulin resistance [32].

### 2.5. Immunoblot Analysis

Brain cortices were thawed on ice, and tissue samples (5–10 mg) were lysed for 30 min in RIPA buffer (50 mM Tris-HCl, pH 7.5; 250 mM NaCl; 5 mM EDTA; 5 mM EGTA; 1 mM dithiothreitol; 0.1% Triton X-100; 0.1% SDS; 1% deoxycholate; 1% Tergitol-type NP40) supplemented with a protease inhibitor cocktail (Complete Protease Inhibitor, Roche Applied Science). Lysates were homogenized using a Dounce homogenizer, followed by sonication. After centrifugation at 14,000× *g* for 10 min at 4 °C, total protein concentrations were determined in the supernatant using the Bradford assay (Bio-Rad, Billerica, MA, USA). Protein samples (40 μg) were denatured at 95 °C for 5 min and separated on 4–15% SDS-PAGE gels. All samples were randomly assigned to gels. Total protein content was visualized via stain-free imaging under UV light (Bio-Rad), and proteins were transferred onto nitrocellulose membranes (Whatman). Membranes were blocked with blocking buffer (5% non-fat dry milk, 0.2% Tween-20 in 1X Tris-buffered saline (TBST); Bio-Rad) and incubated overnight at 4 °C with the following primary antibodies: anti-HSP60 (Heat Shock Protein 60), anti-GSTP1 (Glutathione S-transferase P), anti-Actin, anti-SOD1 (Superoxide Dismutase 1), anti-SOD2 (Superoxide Dismutase 2), anti-catalase, anti-aconitase, anti-FLC (Ferritin Light Chain), anti-FHC (Ferritin Heavy Chain), anti-NQO1 (NAD(P)H dehydrogenase [quinone] 1), and anti-OPA1 (see antibody details in Table 1 below). The next day, membranes were washed with TBST and incubated for 1 h at room temperature with horseradish peroxidase-conjugated secondary antibodies (1/10,000; Abcam, Cambridge, UK). After additional washes, protein detection was performed using chemiluminescence with a Bio-Rad ChemiDoc MP imaging system. Band intensities were quantified using ImageLab 6.1 software (Bio-Rad).

### 2.6. Measurement of Aconitase Activities

Aconitase activity was measured using the Aconitase-340 assay kit (BIOXYTECH, OxisResearch, Foster City, CA, USA), following the manufacturer’s instructions. Tissue samples ranging from 5 to 15 mg were used for each assay. Absorbance of the chromophore was recorded at 340 nm using a V630 spectrophotometer (Jasco, Tokyo, Japan).

### 2.7. Protein Level Score

The score for the oxidative metabolism of both groups (WT and OPA1^+/−^ mice) was defined with a weight value, where 0 corresponds to the lowest and 1 to the highest relative quantity value for each protein (Table 2). A radar representation was used to present the different analyses of each protein for the means of the two groups (Figure 4h).

### 2.8. Statistical Analysis

**Figure 1:**
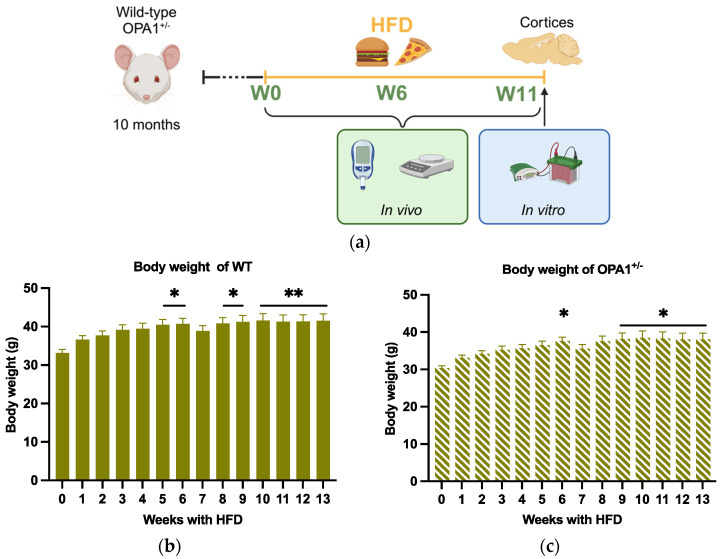
Schematic of experimental design and body weight evolution. (**a**) At 10 months, WT mice and OPA^+/−^ mice were fed with HFD at 10 months of age for 3 months. Metabolic parameters were measured before HFD (W0) and 6 weeks (W6) and 11 weeks (W11) after HFD (created in https://BioRender.com). Finally, all mice were sacrificed at 13 months of age, 3 months after HFD, for biochemical assays of the cortices. (**b**,**c**) Body weight of WT and OPA1^+/−^ mice fed with HFD for 3 months. n = 9–12 mice per group (WT and OPA1^+/−^ fed with HFD) * *p* < 0.05 and ** *p* < 0.01: significantly different from the W0 of WT mice using Tukey’s multiple comparison post hoc test.

**Figure 2:**
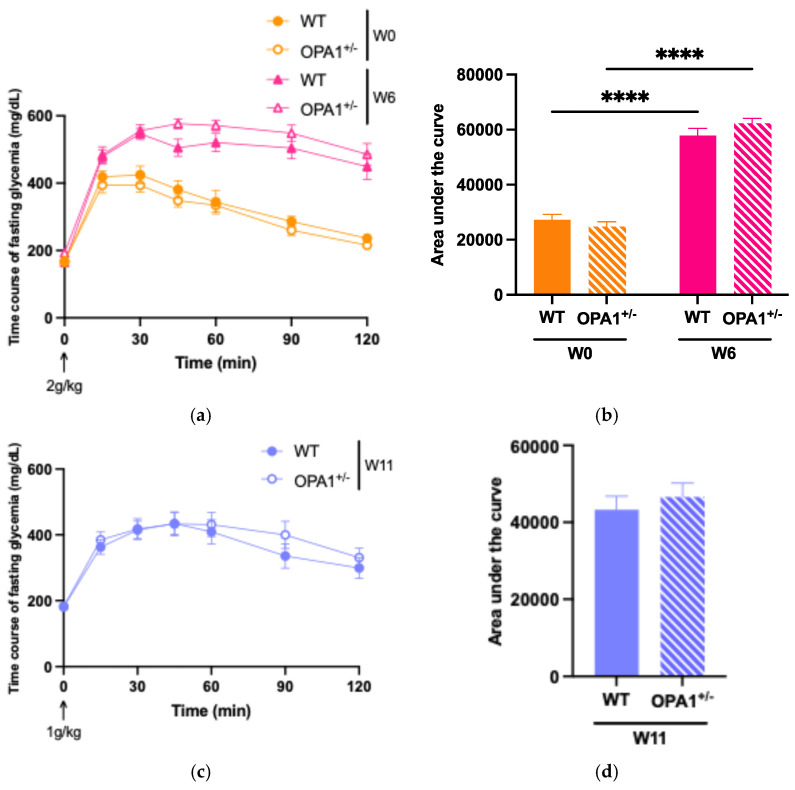

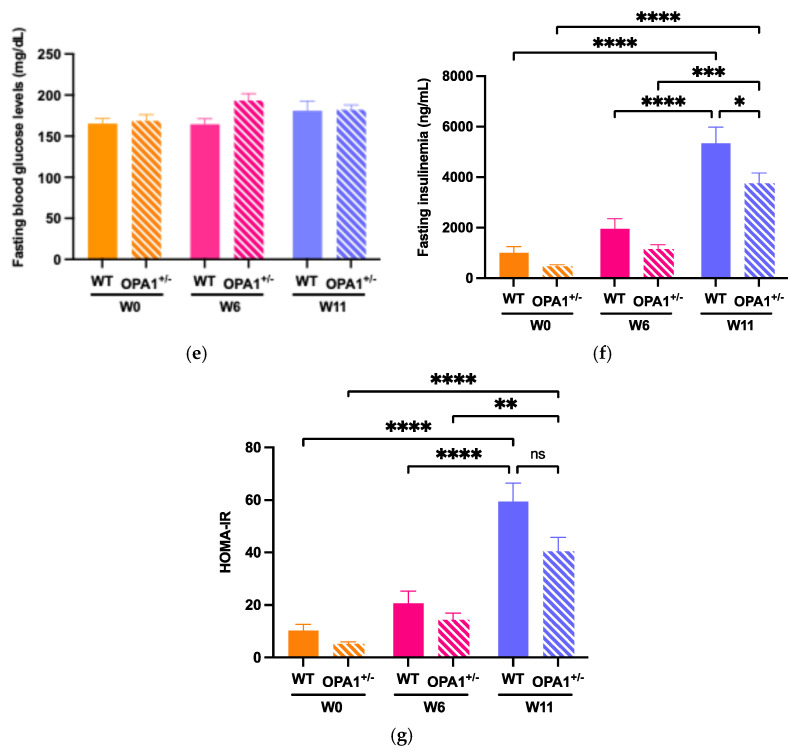
Validation of high-fat diet in both groups. (**a**,**b**) Time course of fasting glycemia during the intraperitoneal glucose tolerance test in response to glucose administration (2 gr/kg; i.p.) and mean ± SEM of Area Under the Curve (AUC) as an index of glucose tolerance before HFD (W0) and after 6 weeks with HFD (W6) in WT and OPA1^+/−^ mice. (**c**,**d**) Time course of fasting glycemia during the intraperitoneal glucose tolerance test in response to glucose administration (1 gr/kg; i.p.) and mean ± SEM of Area Under the Curve (AUC) as an index of glucose tolerance after 11 weeks with HFD (W11) in WT and OPA1^+/−^ mice. (**e**) Fasting blood glucose levels in WT and OPA1^+/−^ fed with HFD, (**f**) fasting plasma levels of insulin in WT and OPA1^+/−^ fed with HFD, and (**g**) HOMA index values (HOMA-IR) in WT and OPA1^+/−^ fed with HFD. n = 9–12 mice per group (WT and OPA1^+/−^ fed with HFD). (**a**–**c**,**e**–**g**) * *p* < 0.05, ** *p* < 0.01, *** *p* < 0.001 and **** *p* < 0.0001 (with two-way ANOVA): significantly different from the corresponding group of WT mice using Tukey’s multiple comparison post hoc test. (**d**) * *p* < 0.05 and ** *p* < 0.01 (with Student’s *t*-test *p*) significantly different from the corresponding group of WT mice.

**Figure 3:**
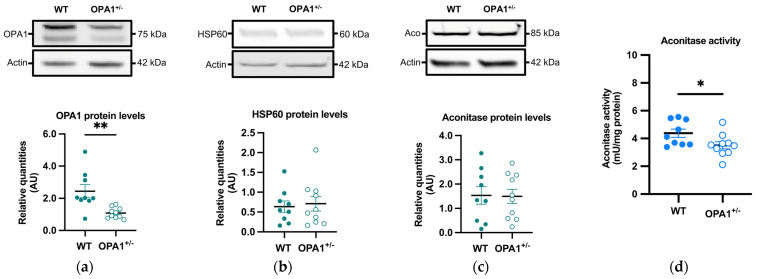
Mitochondrial proteins and aconitase activity in WT and OPA1^+/−^ mice fed with HFD. (**a**–**c**) Representative immunoblots of OPA1, HSP60, and aconitase protein levels in the cortex in both groups. (**d**) Aconitase enzymatic activity (mU/mg of protein) in the cortex in both groups. Data are mean ± SEM. n = 9–10 mice per group (WT and OPA1^+/−^ fed with HFD) * *p* < 0.05 and ** *p* < 0.01 with Student’s *t*-test *p*. * *p* < 0.05 and ** *p* < 0.01: significantly different from the corresponding group of WT mice.

**Figure 4:**
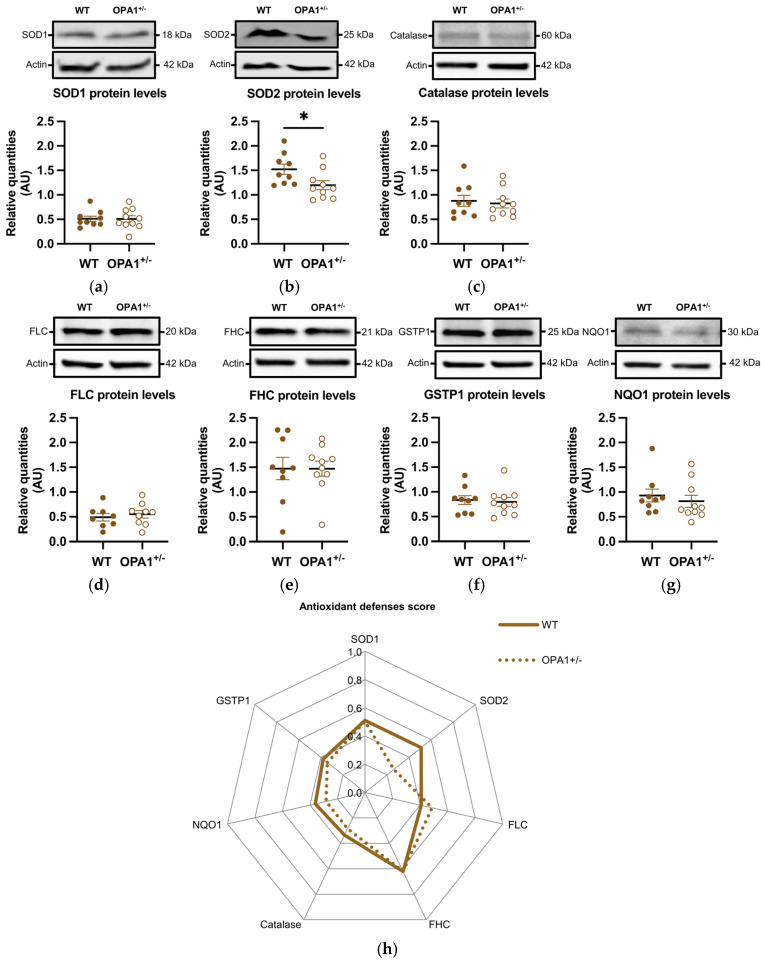
HFD induces a decrease in mitochondrial antioxidant defenses in OPA1^+/−^ mice fed with HFD. (**a**–**g**) Representative immunoblots of SOD1, SOD2, Catalase, GSTP1, FHC, FLC, and NQO1 protein levels in the cortex. (**h**) A representation of each protein quantification based on an average score (0 to 1) for both groups. Data are mean ± SEM. n = 8–10 mice per group (WT and OPA1^+/−^ fed with HFD) * *p* < 0.05 with Student’s *t*-test *p*. * *p* < 0.05: Significantly different from the corresponding group of WT mice. For (**g**), a Mann–Whitney test was applied.

In vivo experiments between WT and OPA1^+/−^ mice were statistically treated using a two-way analysis of variance (ANOVA) with a Tukey’s multiple comparisons post hoc test for body weight (Figure 1b,c) and metabolic measurements (Figure 2). In vitro experiments were statistically treated with Student’s paired t-test because of the systematic comparison between WT and OPA1^+/−^ mice, and the normal distribution was verified (Figure 3 and Figure 4a–h). NQO1 (Figure 4g) protein levels between WT and OPA1^+/−^ mice were determined using Student’s nonparametric test, as the data did not follow a normal distribution (Mann–Whitney test). *p* values * *p* < 0.05, ** *p* < 0.01, and *** *p* < 0.001 were considered statistically significant.

## 3. Results

### 3.1. Effect of Nutritional Challenge on Mouse Body Weight

In order to study the influence of metabolic disturbances induced by the diet as a challenge on OPA1 haploinsufficiency, both WT and OPA1^+/−^ 10-month-old mice were fed with a HFD (diet D12451) for 3 months (Figure 1a). The metabolic parameters were assessed before HFD exposure (W0), and then at six-week intervals (W6 and W11) up to 13 months. At the end of this longitudinal study, all mice were sacrificed (at 13 months old) for molecular assays of the cortices (Figure 1a).

We first observed that WT and OPA1^+/−^ mice gained weight similarly throughout the HFD. The two strains did not present any statistical differences between them, either in their weight before HFD or during the 13 weeks of the experiment. For the WT strain, we observed a statistical increase in bodyweight 5 weeks after the beginning of the diet (Figure 1b). For the OPA1^+/−^ mice, a statistical increase in body wight was detected 6 weeks after the beginning of the experiment (Figure 1c). The small drop in body weight at the seventh week, which is certainly an artifact, is not statistically relevant for either group (Figure 1b,c).

### 3.2. Assessing the High-Fat Diet’s Efficacy in Both WT and OPA1^+/−^ Mice

To evaluate glucose tolerance in both WT and OPA1^+/−^ mice, we administered an intraperitoneal glucose injection and measured fasting plasma glucose concentrations before and after 6 weeks and 11 weeks of HFD feeding (Figure 2). At six (Figure 2a,b) and eleven (Figure 2c,d) weeks of high-fat diet, WT and OPA1^+/−^ mice both showed higher plasma glycemia than the baseline value measured at T0. It is noteworthy that OPA1^+/−^ mice have similar fasting plasma glucose levels to WT mice (Figure 2e).

To assess insulin resistance in WT and OPA1^+/−^ mice, we measured plasma insulin concentrations (Figure 2f) and calculated the Homeostatic Model Assessment of Insulin Resistance (HOMA-IR) (Figure 2g) before and after HFD exposure. We found that 11 weeks of HFD produced a significant increase in plasma insulin levels in both groups compared to W0 (Figure 2f), resulting in elevated HOMA-IR values (Figure 2g), but no differences were detected between genotypes.

Overall, our results indicate that 11 weeks of a HFD impaired glucose tolerance associated with insulin resistance and that the genetic inactivation of OPA1 slightly decreased fasting insulinemia, but no other metabolic parameters.

### 3.3. Mitochondria and Reactive Oxygen Species in Both WT and OPA1 Mice Fed a HFD

OPA1 and HSP60, two mitochondrial proteins, were measured by immunoblotting in OPA1^+/−^ mice fed a HFD. As expected, a significant reduction (approximately 55%) in OPA1 levels was observed in OPA1^+/−^ mice compared to their WT littermates (Figure 3a), while there was no discernible difference in HSP60 levels between the two groups (Figure 3b). The decrease in OPA1 quantity thus did not result in a change in mitochondrial biomass.

We then evaluated the quantity and activity of the aconitase, an enzyme involved in the tricarboxylic acid cycle, through immunoblotting and spectrophotometry, respectively. The sensitivity of aconitase to oxidation arises from damage to its FeS core, making the inhibition of its activity a commonly employed indicator of elevated mitochondrial ROS generation [33,34]. Aconitase quantity showed no significant variation between both groups (Figure 3c), but its activity was significantly lower in OPA1^+/−^ mice compared to WT mice (Figure 3d).

### 3.4. Antioxidant Defenses in Both WT and OPA1^+/−^ Mice Fed a HFD

We then estimated the expression of various antioxidant defense genes, such as SOD1 and SOD2, catalase, ferritins (including both the ferritin light chain and ferritin heavy chain), GSTP1, and NQO1. The genes coding these proteins are targets of the NRF2 transcription factor, which is activated and translocated to the nucleus in response to oxidative stress. The quantity of SOD1 remained unchanged (Figure 4a), while the quantity of SOD2 was significantly reduced in OPA1^+/−^ mice fed with a HFD compared to WT exposed to the same diet (Figure 4b). Furthermore, the levels of the other oxidative metabolism proteins showed no significant differences between the two groups (Figure 4c–g). Figure 4h shows an alternative representation of protein quantification based on an average score. Each raw value of relative protein quantity was scored with a weight value of 0 (low value) or 1 (high value) in order to highlight the protein expression profile of each group (as described in Section 2 and Table 2). The difference between the groups was clearly observed in the expression of SOD2.

## 4. Discussion

Our work addressed the responses of WT and OPA1^+/−^ mice to a HFD in order to determine whether metabolic remodeling induced by the diet can be a modifying factor for DOA.

### 4.1. A HFD Induces a Normal Response in OPA1^+/−^ Mice

We first monitored the response of OPA1^+/−^ mice to fat food imbalance. In the HFD condition, we noticed that the weight of OPA1^+/−^ mice increased during the first three months and then stabilized. The same dynamics were observed for WT mice. Moreover, we did not identify a significant difference between both groups in terms of weight.

We also reported that HFD consumption for 11 weeks in both OPA1^+/−^ and WT mice induced signs of peripheral insulin resistance, as shown by a significant increase in HOMA-IR relative to the same animals tested earlier (i.e., before or after 6 weeks of HFD exposure). Confirming this hypothesis, glucose intolerance was detected in mice fed a HFD for 6 weeks compared to the pre-exposure condition. We anticipate that such a difference is aggravated over time, thereby reinforcing the hypothesis of insulin resistance after 11 weeks of HFD. It is, however, noteworthy that OPA1^+/−^ and WT mice display the same ability to regulate glucose overload, suggesting that the partial genetic inactivation of OPA1 has no effect on peripheral glucose metabolism. Impairments in HOMA-IR are reminiscent of aging, notably on peripheral metabolism [35].

### 4.2. A HFD Induces a Pro-Oxidant State in OPA1^+/−^ Mice

A HFD has been shown to lead to oxidative stress in several tissues, including the brain [29,30], so we investigated this in the brain of OPA1^+/−^ mice. This question was further supported by the relationship between insulin resistance and oxidative mechanism at the periphery [36] and by recent findings providing some evidence that insulin resistance plays a direct role in the brain [35,36,37,38].

First, we proved that aconitase activity was reduced in cortices of HFD-exposed OPA1^+/−^ mice when compared to WT mice and thus could conclude that the production of ROS is higher in a situation of haploinsufficiency in protein OPA1 in mice fed a HFD. Aconitase was indeed previously shown to be highly sensitive to oxidation due to a damaged FeS core and the inhibition of its activity, which are routinely used as a signature of increased mitochondrial ROS production [26,33,39]. This result is in agreement with the data that we collected in vitro using cortical neurons in primary culture and HeLa cells [15,26] and in vivo in cortices of OPA1^+/−^ mice fed a normal diet [25,26].

However, while ROS levels are more important in the cortices of HFD OPA1^+/−^ mice than in WT mice, SOD2 protein levels are lower. SOD2 is an antioxidant enzyme located in the mitochondrial matrix which detoxifies the superoxide anion. This suggests that the antioxidant response is overwhelmed in cortices submitted to a double stressor, i.e., a lack of OPA1 and a HFD. Interestingly, we previously demonstrated that in cortices of OPA1^+/−^ mice fed with a normal diet, the levels of antioxidant defenses were similar when compared to WT mice [25,26]. As ROS levels were also higher in the mutated mice fed a normal diet, this suggests that, in this case, antioxidant defenses are not fully overwhelmed. Thus, metabolic stress increases the redox imbalance in cortices of OPA1^+/−^ mice, reinforcing the idea that a HFD constitutes a supplemental oxidative stress that would be enough to prompt an oxidative stress process.

### 4.3. HFD Redox Imbalance, Neuronal Plasticity, and Cognition

Low mRNA expression of SOD2 in the frontal lobes and in the liver was also demonstrated in obese rats fed with a HFD [40,41]. Interestingly, treatment with a food supplement, including the antioxidant curcumin, rescued the levels of SOD2 and induced a decrease in body fat, glucose intolerance, and metabolic inflexibility [40]. In the same study, a HFD was also shown to decrease spine density in the pre-frontal cortex, associated with deficits in spatial and working memory, which were improved by the addition of a food supplement [40]. Of note, during the revision process, a study has thoroughly reviewed the significant impacts of high-fat/high-sugar diets on cognition and neuronal plasticity [42]. Accordingly, we recently demonstrated that reversible early impairments in hippocampus-dependent spatial memory attributable to defects in hippocampal adult neurogenesis occurred in OPA1^+/−^ mice [43]. It would thus be interesting to investigate whether a HFD worsens these defects and to study the impact of HFDs on mood, since we did not observe this type of disorder with a normal diet. Studies have indeed shown that short-term exposure to a high-fat diet can impair cognitive functions in mice. As a matter of fact, cognitive impairment was observed in juvenile mice fed a 60% HFD for 1 or 3 weeks. This impairment is linked to increased blood–brain barrier permeability and neuroinflammation, which are potential triggering events for cognitive deficits [44]. In the same way, short-term high-fat diets have been associated with increased apoptosis and neuroinflammation in distinct brain regions of mice. This includes the activation of inflammatory signaling proteins and glial cells, which are indicative of stress response pathways such as MAPK signaling [45]. These results may be correlated with human trajectories. Emerging evidence indicates that neurodegenerative diseases might be directly associated with neuroinflammation, including Alzheimer’s disease, Parkinson’s disease, and Amyotrophic lateral sclerosis [46]. Moreover, clinical evidence suggests that HFD consumption influences not only cognition but also emotional processes [47].

## 5. Conclusions

While the main role of the redox imbalance in the disturbance associated with a HFD is well documented [29,30], we demonstrated here that a HFD may influence the expressivity of DOA, a mitochondrial-linked neurodegenerative disease, though aggravation of the redox imbalance. We have thus identified the first environmental modifying factor for this disease. Interestingly, we have previously shown that antioxidant defenses are highly variable in DOA patients, with a more or less drastic SOD1 and SOD2 downregulation. It will therefore be interesting to investigate the dietary habits of DOA patients to determine if the severity and progression of the disease may be correlated to a diet too rich in fat or sugar.

## Figures and Tables

**Table 1:** Dilutions and references of antibodies used for immunoblotting.

Antibody	Dilution	Reference
anti-HSP60	1/3000	Sigma-Aldrich, St. Louis, MI, USA; H-3524
anti-GSTP1	1/2000	Oxford Biochem. Research, Oxford, UK
anti-Actin	1/10,000	Millipore, Burlington, MA, USA; 04-1040
anti-SOD1	1/2000	Abcam, Cambridge, UK; ab51254
anti-SOD2	1/2000	Abcam, Cambridge, UK; ab68155
anti-catalase	1/800	Abcam, Cambridge, UK; ab16731
anti-aconitase	1/500	Abcam, Cambridge, UK; ab83528
anti-FLC	1/1000	Abcam, Cambridge, UK; ab69090
anti-FHC	1/1000	Abcam, Cambridge, UK; ab183781
anti-NQO1	1/1000	Abcam, Cambridge, UK; ab28947
anti-OPA1	1/3000	BD bioscience, San Jose, CA, USA; 612607

**Table 2:** Oxidative metabolism score from relative quantity values of SOD1, SDO2, FLC, FHL, Catalase, NQO1, and GSTP1 protein levels. (**a**) Minimum and maximum relative quantities for each protein in all WT and OPA1 mice, and associated weight values. (**b**) The oxidative metabolism score of both groups (WT and OPA1^+/−^ mice) was defined with a weight value, where 0 corresponds to the lowest and 1 to the highest relative quantity value for each protein.

**(a)**		**Relative Quantities (AU)**													
Proteins		SOD1		SOD2		FLC		FHC		Catalase		NQO1		GSTP1	
Minimum		0.141		0.895		0.180		0.192		0.520		0.390		0.467	
Maximum		0.871		2.102		0.939		2.255		1.588		1.879		1.433	
Weight		1		1		1		1		1		1		1	
**(b)**	**#mice**	**Relative Quantities (AU)**							**Weighted Score**						
		**SOD1**	**SOD2**	**FLC**	**FHC**	**Catalase**	**NQO1**	**GSTP1**	**SOD1**	**SOD2**	**FLC**	**FHC**	**Catalase**	**NQO1**	**GSTP1**
WT	728	0.871	2.102	0.600	1.422	0.520	1.133	0.846	1	1	0.553	0.596	0	0.499	0.393
	755	0.323	1.858	0.513	2.071	0.645	0.583	0.873	0.250	0.798	0.438	0.911	0.117	0.130	0.420
	769	0.444	1.151	0.454	2.255	0.835	0.599	0.819	0.415	0.213	0.361	1	0.295	0.140	0.365
	771	0.561	1.683	0.319	1.486	0.834	0.840	0.550	0.576	0.654	0.184	0.627	0.294	0.302	0.086
	772	0.526	1.637	0.191	2.249	1.588	0.819	1.332	0.528	0.615	0.014	0.997	1	0.288	0.896
	777	0.402	1.410	0.360	0.800	1.145	0.906	1.113	0.358	0.427	0.237	0.294	0.585	0.347	0.668
	785	0.643	1.367	0.884	1.505	0.571	0.880	0.567	0.688	0.391	0.927	0.636	0.048	0.329	0.103
	788	0.443	1.235	0.600	1.292	0.654	0.724	0.869	0.415	0.282	0.553	0.533	0.125	0.224	0.416
	789	0.404	1.136	-	0.192	1.112	1.879	0.533	0.361	0.200	-	0	0.554	1	0.069
	Average	0.513	1.509	0.490	1.475	0.878	0.929	0.834	0.510	0.509	0.408	0.622	0.336	0.362	0.379
OPA1^+/−^	725	0.427	1.792	0.519	1.613	0.522	0.682	0.576	0.393	0.744	0.446	0.689	0.002	0.196	0.113
	726	0.141	1.058	0.594	1.961	0.550	0.644	0.928	0	0.136	0.545	0.857	0.029	0.171	0.477
	727	0.553	1.129	0.389	2.078	0.705	0.390	0.778	0.566	0.195	0.275	0.914	0.173	0	0.321
	743	0.364	1.132	0.344	1.665	0.623	0.544	0.711	0.305	0.197	0.217	0.714	0.097	0.103	0.253
	753	0.713	0.895	0.180	1.352	0.933	1.050	0.734	0.785	0	0	0.562	0.387	0.443	0.276
	754	0.520	1.303	0.939	1.482	1.389	1.354	1.433	0.519	0.338	1	0.625	0.813	0.648	1
	756	0.685	1.721	0.603	1.360	1.235	0.599	0.897	0.745	0.685	0.557	0.566	0.670	0.141	0.445
	770	0.864	1.208	0.763	1.697	0.809	1.566	0.530	0.991	0.260	0.767	0.729	0.270	0.790	0.065
	784	0.393	0.946	0.615	1.174	0.812	0.711	0.911	0.346	0.043	0.573	0.476	0.274	0.216	0.460
	787	0.414	0.921	-	0.339	0.667	0.585	0.467	0.375	0.022	-	0.071	0.138	0.131	0.00
	Average	0.507	1.211	0.549	1.472	0.824	0.812	0.797	0.503	0.262	0.487	0.620	0.285	0.284	0.341

## Data Availability

Data are contained within the article.

## Abbreviations

The following abbreviations are used in this manuscript:

DOA	Dominant Optic Atrophy
AUC	Area Under the Curve
TBST	0.2% Tween-20 in 1X Tris-Buffered Saline
FHC	Ferritin Heavy Chain
FLC	Ferritin Light Chain
GSTP1	Glutathione S-Transferase P
HFD	High-Fat Diet
HOMA-IR	Homeostasis Model Assessment-Estimated Insulin Resistance
HSP60	Heat Shock Protein 60
ipGTT	intraperitoneal Glucose Tolerance Test
LHON	Leber Hereditary Optic Neuropathy
MRC1	Mitochondrial Respiratory Complex 1
NQO1	NAD(P)H Dehydrogenase [Quinone] 1
OPA1	OPtic Atrophy 1
ROS	Reactive Oxygen Species
SOD1	Superoxide Dismutase 1
SOD2	Superoxide Dismutase 2
WT	Wild Type
